# Role of human papillomavirus and cell cycle-related variants in squamous cell carcinoma of the oropharynx

**DOI:** 10.1016/S1674-8301(10)60047-4

**Published:** 2010-09

**Authors:** Guojun Li, Zhigang Huang, Xingming Chen, Qingyi Wei

**Affiliations:** a Department of Head and Neck Surgery, The University of Texas M. D. Anderson Cancer Center, Houston, Texas 77030-4095, USA; b Department of Epidemiology, The University of Texas M. D. Anderson Cancer Center, Houston, Texas 77030-4095, USA; c Department of Otolaryngology Head and Neck Surgery, Beijing Tongren Hospital, Capital Medical University, Key Laboratory of Otolaryngology Head and Neck Surgery, Ministry of Education, Beijing Institute of Otolaryngology, Beijing 100730, China.; d Department of Otolaryngology Head and Neck Surgery, Peking Union Medical College Hospital, Peking Union Medical College and Chinese Academy of Medical Sciences, Beijing 100005, China.

## EPIDEMIOLOGY OF SQAMOUS CELL CARCINOMA OF THE OROPHARYNX (SCCOP)

SCCOP is a subset of squamous cell carcinoma of the head and neck and is characterized by aggressive local tumor growth that requires morbid local-regional therapies. It has a moderately high recurrence rate, common medical comorbidities, and a high frequency of second primary tumors[1]. It is estimated that in the USA approximately 9,000 new cases of SCCOP were diagnosed and 2,110 deaths would result from these cancers in 2006[2]. The median age at diagnosis is approximately 60 y, and this disease occurs more frequently in men (-75%) than in women[2]. For all stages combined, the 5-year relative survival rate from SCCOP is approximately 45% because the majority (-75%) of SCCOP are initially diagnosed at a late stage. The overall 5-year survival rate was virtually unchanged through the 1970s and 1980s, but more recent data shows that, unlike most head and neck cancers, 5-year survival rates for SCCOP have significantly improved in the recent decade[3] due to either a more widespread adoption of chemo-radiation therapies for SCCOP[4],[5] or changing trends in the etiology of SCCOP and its inherent aggressiveness.

Studies utilizing the Surveillance, Epidemiology, and End Results over the past 3 decades show that oral cavity, laryngeal and hypopharyngeal cancers are all in significant decline in incidence, which mirrors the decline in the prevalence of smoking in the USA[3],[6],[7]; however, the incidence of SCCOP has been stagnant or increasing for the past 3 decades[3],[6]. This may be due to an increase of HPV infection, which is supported by rising HPV seroprevalence in Western populations over the past 30 y[8] and changing sexual practices in these populations[9]–[11]. The increase in HPV16 prevalence has been recently reported in studies with human tumor SCCOP specimens[12],[13]. The prevalence of HPV16 in 203 archival SCCOP specimens increased from 23% in 1970s to 28% in the 1980s, 57% in the 1990s, and 68% in the 2000s[12]. During the same period, the prevalence of smoking dramatically declined in this population, leading to the suggestion that an increasing incidence of SCCOP is associated with increasing HPV16 prevalence. This is also supported by data from Colorado, showing that the increasing incidence of SCCOP is linked to increasing prevalence of oncogenic HPV[13]. In this study, the incidence rates of SCCOP in both Colorado and the USA increased between the periods from 1980 to 1990 and from 1991 to 2001, while the rate of HPV-positive oropharynx cases increased from 33% in the 1980s to 70% in the 1990s and 82% in the 2000s[13]. Additionally, for the recent decade, the incidence of SCCOP including base of tongue and tonsil carcinomas in patients under the age of 45 y has actually increased, with a 4-fold increase for tonsil cancers and 1.7-fold for base of tongue cancer[6]. It is possible that SCCOP is evolving from primarily a cancer of middle-aged to elderly smokers and drinkers to one of younger to middle aged nonsmokers who have been exposed to HPV16. The quality of life impact of current therapies (chemoradiotherapy) is particularly problematic if SCCOP becomes a disease of younger nonsmokers without tobacco-related medical comorbidities and with longer life expectancies. Understanding susceptibility for and modifying factors of the HPV16 carcinogenic process will facilitate individualized treatment for SCCOP.

## HPV INFECTION, CELL CYCLE VARIANTS, AND SCCOP

### Mechanisms of HPV-associated carcinogenesis

Cell cycle control is crucial for normal growth and differentiation and genome stability by monitoring the order and integrity of cell division events. The Rb pathway is crucial to DNA damage checkpoint function by eliciting G1-phase cell cycle arrest, while the ATM/p53 pathway controls cell cycle progression *via* regulation of several important genes[14] and induces apoptosis or G1 cell-cycle arrest[15]. The loss of function of the ATM/p53 or Rb pathways results in the loss of cell cycle control and checkpoint integrity, allowing unchecked progression through the cell cycle and proliferation, instead of DNA damage repair or damage-induced apoptosis[16].

Integration of HPV DNA into the host-cell genome causes viral DNA interruption. Integration in the region of the E2 gene may cause a disruption in HPV oncogenic E6 and E7 transcriptional control, resulting in an increased expression of E6 and E7, which interact with cell cycle regulators[17]. For example, E6 may bind to p53 of the host cell, p53 degradation *via* the ubiquitination pathway[18],[19], while E7 binds to Rb, resulting in the degradation and release of the E2F transcription factors, which reads to the deregulation of cell cycle control and uncontrolled cell proliferation[20]. Therefore, inactivation of both p53 and Rb by E6 and E7 allows the cell to escape normal cell cycle checkpoints, with resultant cellular transformation and immortalization. Other cofactors such as genetic susceptibility and immune function may also be necessary for the persistence of HPV infection and the modification of HPV associated malignancies.

### Evidence of HPV in SCCOP

While only 10-20% of head and neck cancers are associated with high-risk, oncogenic HPV types, these HPV-associated cases represent a distinct subset of tumors, chiefly SCCOP with distinct epidemiologic, clinical, and molecular characteristics. With the progress in molecular techniques, oncogenic HPV DNA has been consistently detected in approximately 50% of SCCOP and this may be much higher in certain groups of SCCOP, such as those lacking significant tobacco exposures[21]–[25]. For HPV^+^ tumors, HPV16 has been identified in 90-95% of the tumors followed by HPV31, 33 and 18[21],[26]. Additionally, there appear to be dramatic differences in the HPV16^+^ tumor prevalence rates between oral cavity and oropharyngeal tumors, despite common misclassifications of base of tongue and some soft palate cancers as oral cavity tumors, falsely elevating the reported HPV prevalence rates of oral cavity tumors[10],[26],[27]. While it has been suggested that certain subgroups of SCCOP patients are more likely to be HPV16^+^, further study is needed to document the influence of demographic, clinical, and exposure characteristics, as well as potential genetic variants, on HPV16 prevalence rates.

### Association of HPV with risk of SCCOP

The strongest evidence of an association of HPV16 with risk of SCCOP came from molecular epidemiologic studies (HPV16 serologic or tumor DNA status) with a case-control design, showing a range of odds ratios from 3 to 60[10],[23],[26]–[33]. For example, a nested case-control study of 292 cases and 1,568 controls from a prospective Scandinavian cohort of almost 900,000 subjects from whom blood was collected prospectively before cancer diagnosis found that HPV16 seropositivity was significantly associated with a 14.4-fold increased risk of SCCOP[27]. In our pilot study of 120 cases and 120 matched controls, we found that 59% of SCCOP patients were serologically positive for HPV16 compared with only 9% of cancer-free controls, and of the non-smokers with SCCOP 69% were positive[31]. Misclassification of cancer sites, inadequate control for environmental exposure, and heterogeneity in HPV16 detection methodology are serious limitations of the current literature. Other potential confounding factors and sources of heterogeneity that cause inconsistent results should be also taken into consideration in future epidemiological studies.

### Association of genetic alterations of cell cycle regulators with HPV-associated SCCOP

Cell cycle-related genes play a role in modulating cellular DNA repair, cell-cycle control, cell growth and apoptosis. Simultaneous analysis of genetic alterations in both the ATM/p53 and Rb pathways may aid in understanding the distinct mechanisms underlying HPV16^+^ and HPV16^−^ SCCOP. The alteration of genes in both the ATM/p53 and Rb pathways can lead to loss of appropriate tumor suppressor functions. The biological and etiological distinctions between HPV16^+^ and HPV16^−^ SCCOP support the concept of two different mechanisms in the development of SCCOP, the former driven by oncogenic HPV and the latter by tobacco and alcohol. For example, HPV16^+^ SCCOP has been characterized by wild type p53, decreased expression of CCND1 and Rb, upregulation of p16, and presence of HPV16 DNA while HPV16^−^ SCCOP lacks HPV16 DNA and shows mutated p53, overexpresses CCND1 and Rb, and has decreased expression of p16[34]–[37]. While p53 and/or p16 mutations are rare in HPV16^+^ tumors, p53 and p16 alterations, such as mutation or loss of heterozygosity, are common in HPV16^−^ tumors[38]. Here, I briefly summarize some of the relevant findings for some genetic alterations in both pathways.

The presence of p53 mutations and HPV16 positivity of SCCOP appear to be inversely correlated[21],[34],[39]–[41]. p53 alterations caused by both somatic mutations and germline variants have been documented[42],[43]. A common single nucleotide polymorphism (SNP) of p53 at codon 72 in exon 4 results in a substitution of Pro for Arg in the transactivation domain[44]. The common Arg variant allele appears to alter the susceptibility of p53 to oncogenic HPV E6-mediated degradation[45], and this allele was significantly associated with oncogenic HPV infection[46]–[48]. Furthermore, in case-control analyses, the homozygous Arg/Arg genotype has been significantly associated with an increased risk of HPV-associated cancer[45],[49]; however, some studies have not confirmed these findings[50]–[53]. p73, a member of the p53 family, activates the promoters of several p53-responsive genes involved in cell-cycle control, DNA repair, and apoptosis, and p73 inhibits cell growth in a p53-like manner by inducing apoptosis or G1 cell-cycle arrest[15]. Inactivation of p73 by oncogenic HPV E6 appears analogous to that of p53 without modulating their DNA binding activities[54]. p73, unlike p53, is resistant to degradation by HPV16 E6 and can suppress growth and induce apoptosis in HPV16 E6-expressing cells[55]. It is possible that p73 variants could alter the affinity of the E6 protein for p73 and thus alter the risk for HPV16-associated carcinogenesis. The two linked non-coding exon 2 polymorphisms of *p73* at position 4 (G→A) and 14 (C→T) are thought to affect p73 function by altering gene expression, perhaps by altering the efficiency of translational initiation[56]; whether this alters the effect of E6 on p73 is unknown.

MDM2 negatively regulates p53 levels by modulating p53 cellular activity[57]. A significant association between HPV positivity in esophageal carcinoma and overexpression of p53 and MDM2[58],[59] indicates an interplay between HPV infection and these two genes in esophageal carcinogenesis. Moreover, aberrant MDM2 and p53 expression is frequently found in cervical neoplasia and overexpression of MDM2 in invasive carcinomas may protect against HPV E6-induced p53 degradation[60],[61]. Recently, a new SNP G2580T at *MDM2* nucleotide 309 in the promoter region was identified[62]. This polymorphism has been shown to alter p53 expression levels with subsequent attenuation of the p53 pathway[62], but the association of this polymorphism with HPV16 status in SCCOP has not been investigated.

There also appears to be a strong association between HPV positivity and reduced CCND1 expression in tonsil carcinomas[36]. CCND1 promotes transition through the restriction point in the G1 phase of the cell cycle[63]. A polymorphism of *CCND1* exists at codon 242 within the conserved splice donor site of exon 4, modulating the splicing of CCND1 mRNA and causing two transcripts with different half-lives and functional activity[64]. The reduced levels of CCND1 may facilitate the interaction of the HPV16 E7 with Rb, but this has not been documented. It appears that HPV16 E7 binds to Rb, which in turn results in p16 overexpression. p16 subsequently blocks cell-cycle progression by binding to cyclin-dependent kinase 4 and 6 as well as inhibiting cyclin D[65]. Both HPV^+^ cervical cancers and HPV16^+^ head and neck cancers have been shown to overexpress p16[34],[41],[65]–[68], and recently, microarray data has confirmed this association between p16 overexpression and HPV16 positivity in head and neck cancers[69]. Two adjacent polymorphisms (C540G and C580T) of p16 have been identified in the 3' untranslated region of exon 3[70]. Furthermore, the C580T polymorphism co-segregates with the C74A polymorphism of intron 1 of *p16*[70]. Because the C540G polymorphism is associated with low expression of p53 and both polymorphisms have been associated with tumor aggressiveness[70], these two variants may have some functional relevance. Therefore, alterations of these cell cycle regulators in both the ATM/p53 and Rb pathways may jointly alter both cell cycle checkpoints and apoptosis. Genetic variants of these genes could affect inter-individual variations in efficiency of E6 and E7's impact on these pathways that lead to differences in apoptotic capacity, resulting in different rates of both HPV clearance, and such genetic variants could modify susceptibility to HPV infection. Despite considerable studies demonstrated association of polymorphisms in cell cycle related genes in many types of cancer, data are quite conflicting. Moreover, there are no published studies investigating association of the polymorphisms in these genes with HPV16 status and SCCOP outcomes.

### Impact of tumor HPV16 on SCCOP prognosis

It has been suggested that HPV16-associated SCCOP represents a different disease not only on a molecular level but also in clinical outcomes. While some authors have documented improved survival and lower recurrence rates in SCCOP patients with HPV16^+^ tumors[21],[23],[39],[71]–[79], other studies have not found such a prognostic impact[40],[77],[80]–[83]. The suggestion that HPV tumor positivity is a good prognostic marker needs to be viewed critically given that significant confounding is not controlled for in many such studies. The interplay between HPV16 E6 and E7 proteins and cell cycle regulators in the ATM/p53 and Rb pathways may lead to up- or down-regulation of these genes' expression, thus affecting clinical outcomes[23],[36],[58],[73],[74],[78],[84]–[89]. Additionally, it is likely that tobacco-associated SCCOP is more genetically diverse with a multitude of mutations, and as such may have a potential for resistance to radiation therapy.

One of the reasons for improved survival in HPV16^+^ SCCOP patients could be that degradation of p53 is not functionally equivalent to p53 mutations. It is possible that HPV16^+^ tumors with wildtype p53 maintain an intact apoptotic response and thus could be more responsive to radiation, while tobacco-associated SCCOP with mutated p53 may have a degree of radioresistance[86]–[89]. Of course, a major confounder is smoking and HPV16^+^ tumors in smokers may have genetic modifications of a tobacco-associated tumor with mutated p53 and theoretical radioresistance. After stratification by p53 status, HPV16^+^ patients with a p53 mutation had the worst prognosis compared with HPV16^+^ patients with wildtype p53 or HPV16^−^ patients without p53 mutation[90]. In addition, other alterations of cell cycle regulators caused by the interplay with HPV16 may also affect the apparent association between HPV16 and survival. A significant correlation of p16 expression with HPV16 positivity and increased disease-free survival has also been reported[91]. In a recent study, survival and risk of 5-year local recurrence were significantly improved in SCCOP patients with HPV16^+^ and high p16 expression compared with patients with HPV16^−^ and low p16 expression or HPV16^+^ and low p16 expression[92]. This study provides a valuable clue that patient stratification by both HPV16 status and genetic alterations for SCCOP may be needed due to other confounders for prognosis such as smoking in head and neck cancer patients[93].

Because somatic genetic changes of these genes are rare among HPV16^+^ SCCOP tumors and the conflicting data regarding the impact of HPV16 status on prognosis is further confounded by other genetic alterations or confounding factors, genetic polymorphism could play a significant role in person-to-person variability in prognosis. The polymorphisms in likely functional regions of these genes may affect their biological function and potentially interactions with the HPV E7 and E6 proteins. Therefore, such genetic variants could constitute a confounding effect on HPV-related clinical outcomes.

## SUMMARY

By knowing the HPV16 status of SCCOP patients, there may be important prognostic implications and potential influences on current and future treatment and prevention strategies. If these patients do indeed have a significantly better prognosis, it may be possible to reduce the intensity of current treatments in HPV16^+^ SCCOP patients as well as to develop future targeted therapies for such patients for an improved survival and a better quality of life. Additionally, these genetic polymorphisms could define the molecular profile of HPV16^+^ SCCOP and optimize patient stratification for clinical trials of HPV16-targeted therapies, or improved prognostication could facilitate more selective use of local and systemic therapies.
